# Dietary saccharides and sweet tastants have differential effects on colonization of *Drosophila* oocytes by *Wolbachia* endosymbionts

**DOI:** 10.1242/bio.023895

**Published:** 2017-06-08

**Authors:** Moises Camacho, Mailin Oliva, Laura R. Serbus

**Affiliations:** 1 Department of Biological Sciences, Florida International University, 11200 SW 8th St, Miami, FL 33199, USA; 2 Biomolecular Sciences Institute, Florida International University, 11200 SW 8th St, Miami, FL 33199, USA

**Keywords:** Symbiosis, *Wolbachia*, *Drosophila*, Transmission, Oocyte, Titer

## Abstract

*Wolbachia* bacteria are widespread, maternally transmitted endosymbionts of insects. Maintenance of sufficient *Wolbachia* titer in maternal germline cells is required for transmission efficacy. The mechanisms that regulate *Wolbachia* titer are not well understood; however, dietary sucrose was reported to elevate oocyte *Wolbachia* titer in *Drosophila melanogaster* whereas dietary yeast decreased oocyte titer. To further investigate how oocyte *Wolbachia* titer is controlled, this study analyzed the response of *w*Mel *Wolbachia* to diets enriched in an array of natural sugars and other sweet tastants*.* Confocal imaging of *D. melanogaster* oocytes showed that food enriched in dietary galactose, lactose, maltose and trehalose elevated *Wolbachia* titer. However, oocyte *Wolbachia* titers were unaffected by exposure to the sweet tastants lactulose, erythritol, xylitol, aspartame and saccharin as compared to the control. Oocyte size was generally non-responsive to the nutrient-altered diets. Ovary size, however, was consistently smaller in response to all sugar- and sweetener-enriched diets. Furthermore, most dietary sugars administered in tandem with dietary yeast conferred complete rescue of oocyte titer suppression by yeast. All diets dually enriched in yeast and sugar also rescued yeast-associated ovary volume changes. This indicates oocyte colonization by *Wolbachia* to be a nutritionally sensitive process regulated by multiple mechanistic inputs.

## Abstract (precis)

**Summary:** The density of *Wolbachia* infection in *Drosophila* oocytes is significantly increased by dietary sugars, but not sugar alcohols or artificial sweeteners.

## INTRODUCTION

Metazoan organisms are increasingly recognized as communities of prokaryotic and eukaryotic cells. Symbiotic interactions within the collective unit of an organism range from mutualistic to parasitic (Dale and Moran, 2006). Endosymbiotic *Wolbachia* bacteria are unique in that they occupy a wide range of the symbiotic spectrum. *Wolbachia* are *Alphaproteobacteria* that reside within the cells of mites, crustaceans, filarial nematodes (Werren et al., 2008) and approximately 52% of all insect species based on a handful of typing loci (Weinert et al., 2015). At least 470 distinct *Wolbachia* strains have been reported to date (Baldo et al., 2006). Of those, some are reported to provide essential cofactors to the host (Ghedin et al., 2007; Hosokawa et al., 2010; Nikoh et al., 2014), promote host reproduction (Dedeine et al., 2001; Landmann et al., 2011; Starr and Cline, 2002) and protect the host from lethal RNA viruses (Chrostek et al., 2013; Hedges et al., 2008; Martinez et al., 2014; Teixeira et al., 2008). Conversely, the *w*Mel^Pop^ *Wolbachia* variant lyses brain cells and shortens insect lifespan (Min and Benzer, 1997). This positions *Wolbachia* as a uniquely informative system for elucidating the cellular mechanisms of symbiosis.

A consensus requirement for *Wolbachia* success across diverse hosts is robust vertical transmission. Though *Wolbachia* occupy the germline stem cells (GSC) of male and female hosts, removal of the bacteria during spermatogenesis creates a ‘dead end’ with respect to transmission (Bressac and Rousset, 1993; Serbus et al., 2008). Thus, persistence of *Wolbachia* in maternal germline cells is of critical importance for transmission to progeny. In the *Drosophila melanogaster* model system that naturally carries *w*Mel *Wolbachia* (O'Neill et al., 1992; Riegler et al., 2005), the GSC are infected with these bacteria. This ensures that differentiating daughter cells (cystoblasts) inherit *Wolbachia* during mitosis (Ferree et al., 2005; King, 1970; Serbus et al., 2008). While the cystoblast undergoes mitosis to generate an interconnected cyst of 16 germline cells, *Wolbachia* exiting the nearby somatic cell niche also invade the germline cyst (Toomey et al., 2013). After the cyst is coated with a blanket of somatic follicle cells, creating a unit referred to as an egg chamber (King, 1970), additional horizontal invasion events may also occur (Casper-Lindley et al., 2011). *Wolbachia* also replicate to populate the germline cells of the egg chamber, including the oocyte cell that ultimately takes over to form a completed egg (King, 1970; Serbus et al., 2011). Similar germline loading mechanisms are expected to apply to other *Wolbachia-Drosophila* combinations, with differential contributions to germline colonization by GSC loading and horizontal invasion in each case (Toomey et al., 2013).

Maternal transmission relies upon sufficient *Wolbachia* titer within the germline cells. One strategy of *Wolbachia* transmission in embryogenesis is the use of mass action to promote inclusion of bacteria in embryonic germline cells (Veneti et al., 2004)*.* A complementary strategy to facilitate bacterial transmission is through strategic subcellular localization (Breeuwer and Werren, 1990; Hadfield and Axton, 1999; Rasgon and Scott, 2003; Stouthamer et al., 1993; Veneti et al., 2004; Zchori-Fein et al., 1998). In *D. melanogaster*, the host microtubule motor proteins Dynein and Kinesin-1 act sequentially to elevate *Wolbachia* concentration at the oocyte posterior cortex (Ferree et al., 2005; Serbus and Sullivan, 2007). This is followed by association of *Wolbachia* with a cortical mixture of components referred to as pole plasm (Ashburner, 1989; Riechmann and Ephrussi, 2001; Serbus and Sullivan, 2007). This positions the bacteria for envelopment by embryonic germline cells specified by the pole plasm (Ashburner, 1989; Hadfield and Axton, 1999; Serbus and Sullivan, 2007). Maternal *Wolbachia* transmission rates documented in *D. melanogaster* are near 97% in the field (Hoffmann et al., 1998) and 100% in the lab (Turelli and Hoffmann, 1995), indicating this maternal transmission strategy is effective.

The molecular mechanisms that regulate *Wolbachia* titer are not well understood. Body-wide *Wolbachia* titer has been reported to vary up to 180,000-fold in lab-reared offspring of mosquitoes collected from nature (Ahantarig et al., 2008), and 20,000-fold between wild-caught *Drosophila innubila* individuals (Unckless et al., 2009). This titer variation may be due in part to sensitivity to host temperature (Bordenstein and Bordenstein, 2011; Mouton et al., 2006, 2007; Wiwatanaratanabutr and Kittayapong, 2009, 2006), host crowding (Hoffmann et al., 1998; Wiwatanaratanabutr and Kittayapong, 2009), host genetic background (Boyle et al., 1993; Poinsot et al., 1998; Veneti et al., 2004; Serbus et al., 2011) and host age (Tortosa et al., 2010; Unckless et al., 2009).

A set of studies has particularly highlighted the impact of diet on *Wolbachia* titers *in vivo*, implicating roles for dietary cholesterol (Caragata et al., 2013) and other macronutrients (Ponton et al., 2015). It was recently shown that dietary yeast, known to trigger insulin signaling in *Drosophila* (Géminard, 2009 #1357; Teleman, 2010 #1333), suppresses *Wolbachia* titer in developing oocytes (Serbus et al., 2015 #1785). By contrast, dietary sucrose, which is expected to induce insulin resistance in *Drosophila* (Broughton et al., 2010; Morris et al., 2012; Musselman et al., 2011; Norseen et al., 2012; Pasco and Léopold, 2012; Yang et al., 2005), led to elevated oocyte titers (Serbus, 2015 #1785). Understanding how diet affects oocyte *Wolbachia* titer is expected to inform the mechanisms supporting *Wolbachia* colonization of host cells and ultimately, *Wolbachia* transmission. To address the mechanisms underlying *Wolbachia* titer control in oogenesis, an array of structurally diverse dietary sugars and sweet tastants was selected, and their impact on colonization investigated as described below.

## RESULTS

### Dietary sugars elevate oocyte *Wolbachia* titer in *D. melanogaster*

**Fig. 1.**
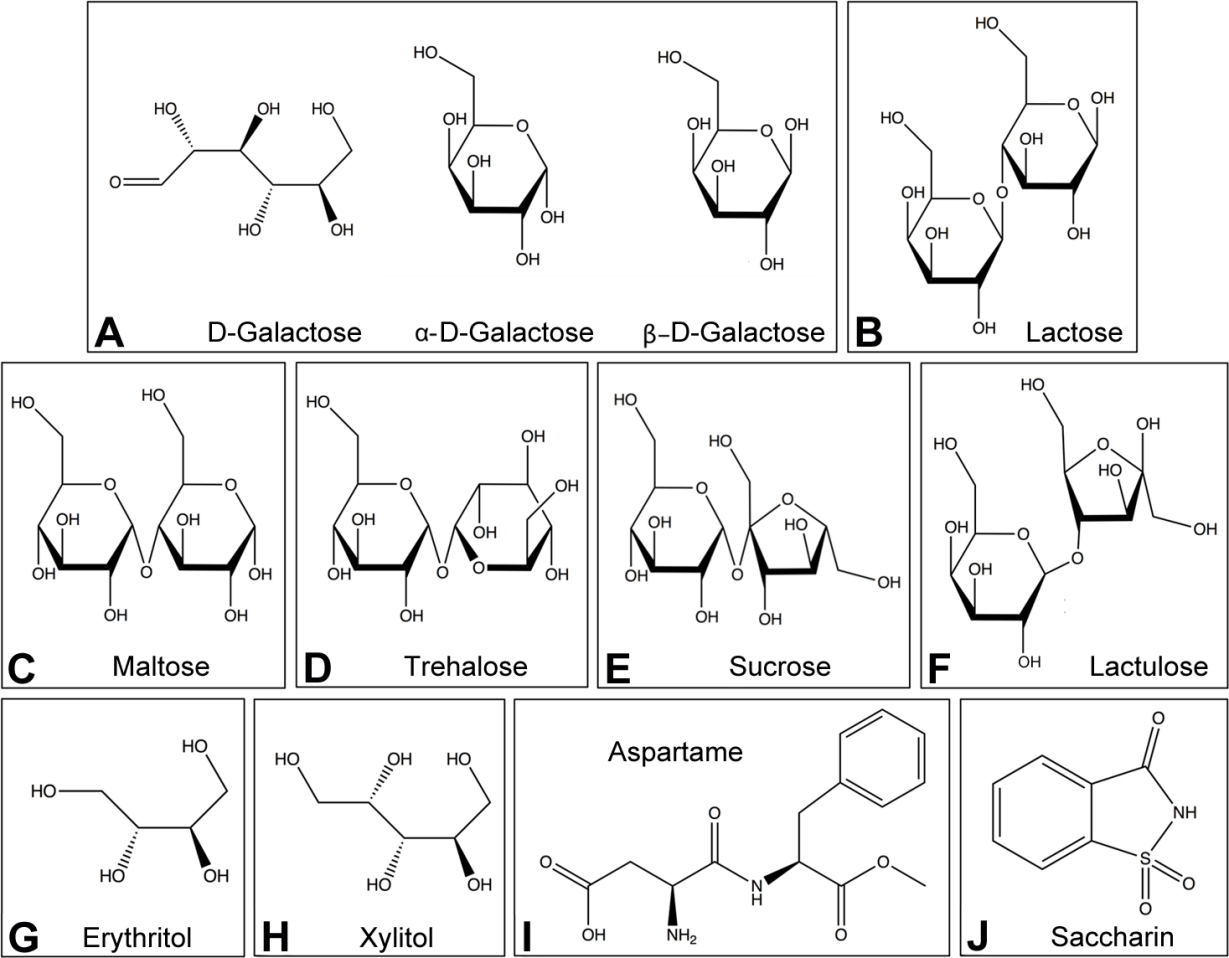
**Structures of the sugars, sugar alcohols and artificial sweeteners used.** Natural saccharides included: (A) D-galactose, shown in open ring, alpha-pyranose and beta-pyranose conformations; (B) lactose; (C) maltose; (D) trehalose; (E) sucrose. Synthetic disaccharide: (F) lactulose. Sugar alcohols: (G) erythritol; (H) xylitol. Artificial sweeteners: (I) aspartame; (J) saccharin.

**Fig. 2.**
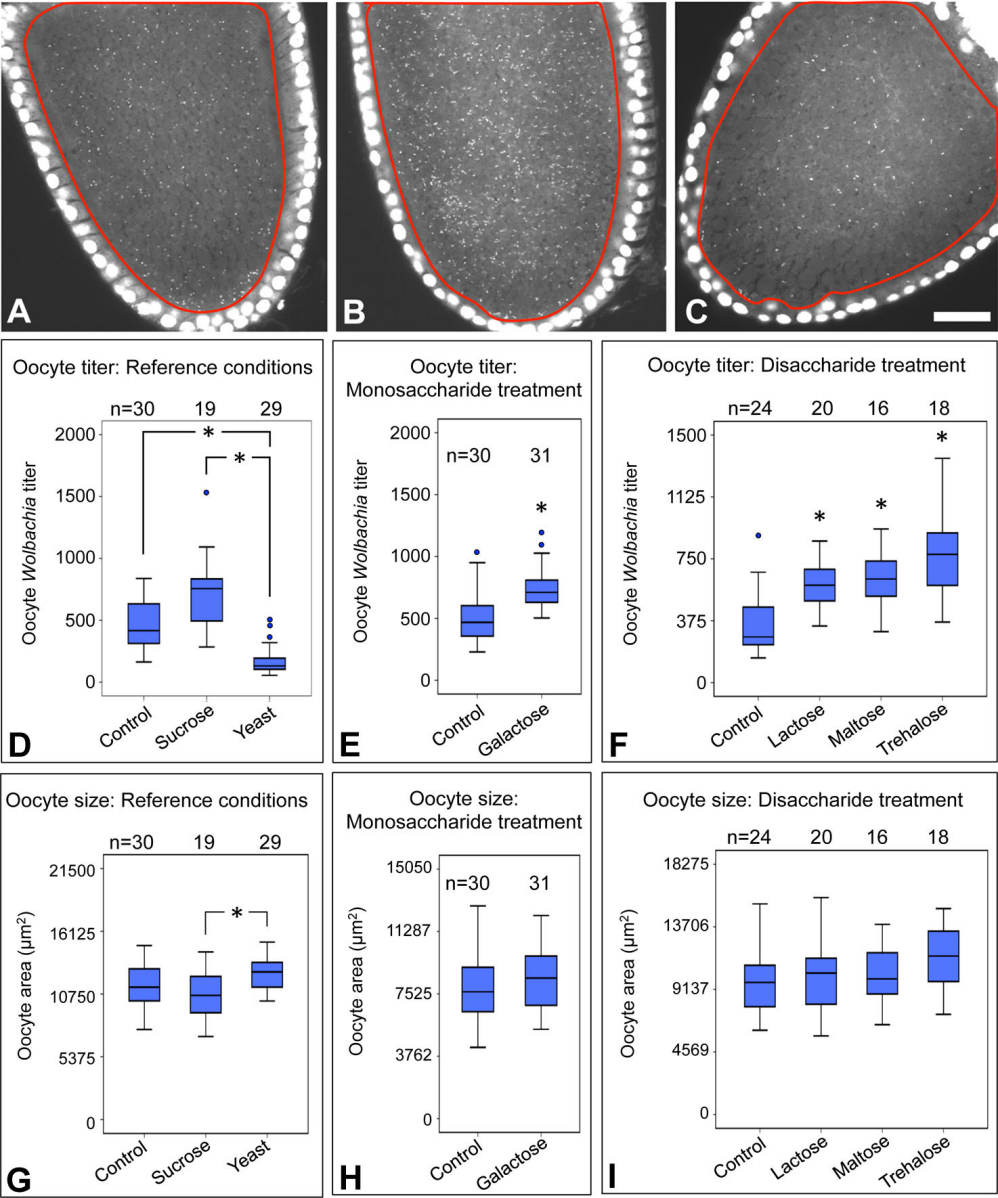
**Effects of dietary sugars on oocyte *Wolbachia* titer and oocyte size.** (A-C) Stage 10 *D. melanogaster* oocytes imaged by confocal microscopy are outlined in red. DNA staining indicates the *Drosophila* DNA as large circles and *Wolbachia* nucleoids as small puncta. Treatment conditions: (A) control food; (B) sucrose-enriched food; (C) yeast-enriched food. Scale bar: 25 μm. (D-I) Graphs indicate the average number of *Wolbachia* nucleoids displayed by single oocyte focal planes. Oocyte *Wolbachia* titer was scored in response to foods enriched with the following nutrients: (D) sucrose and yeast, (E) the monosaccharide galactose, and (F) the disaccharides lactose, maltose and trehalose. Oocyte size was also assessed from the same set of confocal images, to determine the profile of oocyte area for the following nutrient-enriched diets: (G) sucrose and yeast, (H) the monosaccharide galactose and (I) the disaccharides lactose, maltose and trehalose. To collect these data, three biological replicates were performed, with 20 flies dissected per condition per replicate. The sample size (*n*) for all experimental conditions is included in the figure. Median values are displayed as the middle line within each boxplot, and the boxed areas represent the interquartile range. The box whiskers indicate minimal and maximum values of the dataset, except for the outliers which are shown as solid blue circles. Significance is indicated by asterisks, as according to Kruskal–Wallis ANOVA. Significance values by panel are: (D,E) *P<0.001; (F) control vs lactose: *P=0.002, control vs maltose:*P<0.001, control vs trehalose: *P<0.001; (G) oocyte size: sucrose vs yeast: *P=0.004.

Prior results indicated that yeast-enriched food reduces *Wolbachia* titers in oogenesis, whereas sucrose-enriched food elevates oocyte *Wolbachia* titer (Serbus et al., 2015). To confirm this, two-day-old flies were exposed to yeast- and sucrose-enriched food for 3 days (Fig. 1E, Table 1; Table S1). The ovarian tissues were dissected, fixed, stained with propidium iodide and imaged by confocal microscopy. Each punctate nucleoid that is labeled by the DNA stain is interpreted as representing a single bacterium*.* The resulting images suggested overall more *Wolbachia* puncta in the sucrose condition, and fewer *Wolbachia* puncta in the yeast condition (Fig. 2A-C). For finer resolution of oocyte *Wolbachia* titer, *Wolbachia* were quantified from representative oocyte focal planes, and analyzed by Kruskal–Wallis ANOVA. According to these criteria, oocyte *Wolbachia* titer in the yeast-enriched condition was significantly lower than the control [χ^2^(2)=27.3, *P*<0.001] (Fig. 2D). Though higher oocyte *Wolbachia* titers were detected in the sucrose-enriched condition, the values did not differ significantly from the control [χ^2^(2)=15.6, *P*=0.056]. Significant oocyte titer differences were detected between the yeast and sucrose conditions, however, with sucrose exhibiting a 577% higher median titer value than yeast [χ^2^(2)=42.9, *P*<0.001] (Fig. 2D). Overall, this outcome corroborates opposing effects of dietary sucrose and yeast on oocyte *Wolbachia* titer.

**Table 1.**
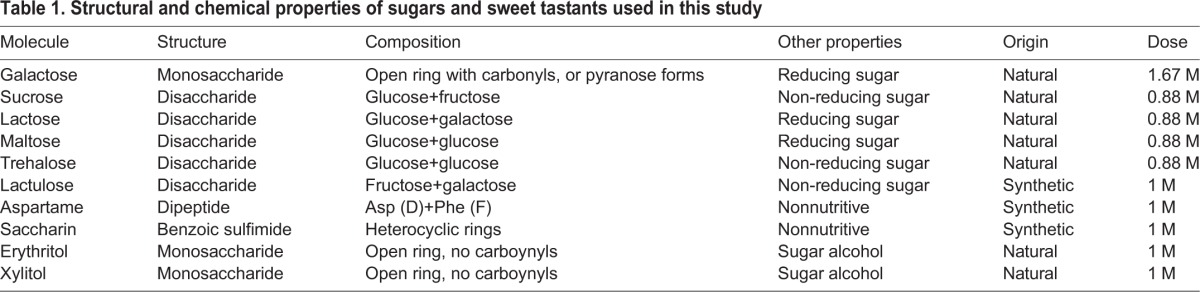
**Structural and chemical properties of sugars and sweet tastants used in this study**

A surprising finding from prior work was that food enriched in the monosaccharide constituents of sucrose, namely glucose and fructose, did not recapitulate high oocyte titer responses analogous to sucrose (Serbus et al., 2015). This raised questions as to whether any monosaccharide is capable of affecting oocyte *Wolbachia* titer. To test this, two-day-old flies were collected and exposed to galactose-enriched food for 3 days (Fig. 1A, Table 1; Table S1). *Wolbachia* quantification indicated that galactose-fed flies carried significantly more *Wolbachia* than control oocytes [χ^2^(1)=18.2, *P*<0.001] (Fig. 2E). This indicates galactose to be the first dietary monosaccharide capable of elevating oocyte *Wolbachia* titer.

To test the extent to which other dietary disaccharides affect oocyte *Wolbachia* titer, flies were exposed to lactose-, maltose- and trehalose-enriched foods (Fig. 1B-D, Table 1; Table S1). These treatments elevated oocyte *Wolbachia* titer, with magnitude increasing from lactose to maltose to trehalose (Fig. 2F). Oocyte *Wolbachia* titers in disaccharide enriched conditions were also identified as significantly different from the control [lactose χ^2^(3)=25.0, *P*=0.002; maltose χ^2^(3)=28.8, *P*<0.001; trehalose χ^2^(3)=39.0, *P*<0.001] (Fig. 2F). This indicates that exposure to a range of disaccharide-enriched diets increases oocyte *Wolbachia* titer.

### Oocyte size is generally non-responsive to sugar-enriched foods

To consider the basis for oocyte titer changes, oocyte size was tested. Specifically, the two-dimensional area of every oocyte image used for titer quantification above was measured. As all sample compression and oocyte focal plane selection were standardized for each experiment, the resulting area values are a proxy estimate for oocyte size. According to this analysis, no significant differences in oocyte area were identified between control and sucrose-enriched conditions [χ^2^(2)=12.2, *P*=0.085], nor control and yeast-enriched conditions [χ^2^(2)=6.6, *P*=0.811] (Fig. 2G). Significance was detected when comparing oocyte area values between sucrose- and yeast-enriched conditions [χ^2^(2)=18.7, *P*=0.004]. The median area of sucrose-treated oocytes was 84% of the yeast condition (Fig. 2G), in contrast to the 577% disparity between median oocyte *Wolbachia* titers in these conditions (Fig. 2D). Oocyte area did not differ significantly between control and galactose-fed oocytes [χ^2^(1)=1.27, *P*=0.26] (Fig. 2H), nor between control, lactose-, maltose-, and trehalose-fed oocytes [χ^2^(3)=6.72, *P*=0.083] (Fig. 2I). Thus, oocyte area did not parallel the significantly higher oocyte *Wolbachia* titer responses to natural saccharides (Fig. 2E,F). This suggests that oocyte size changes are not responsible for sugar-induced increases in oocyte *Wolbachia* titer.

### Ovary size is consistently smaller in response to sugar-enriched diets

**Fig. 3.**
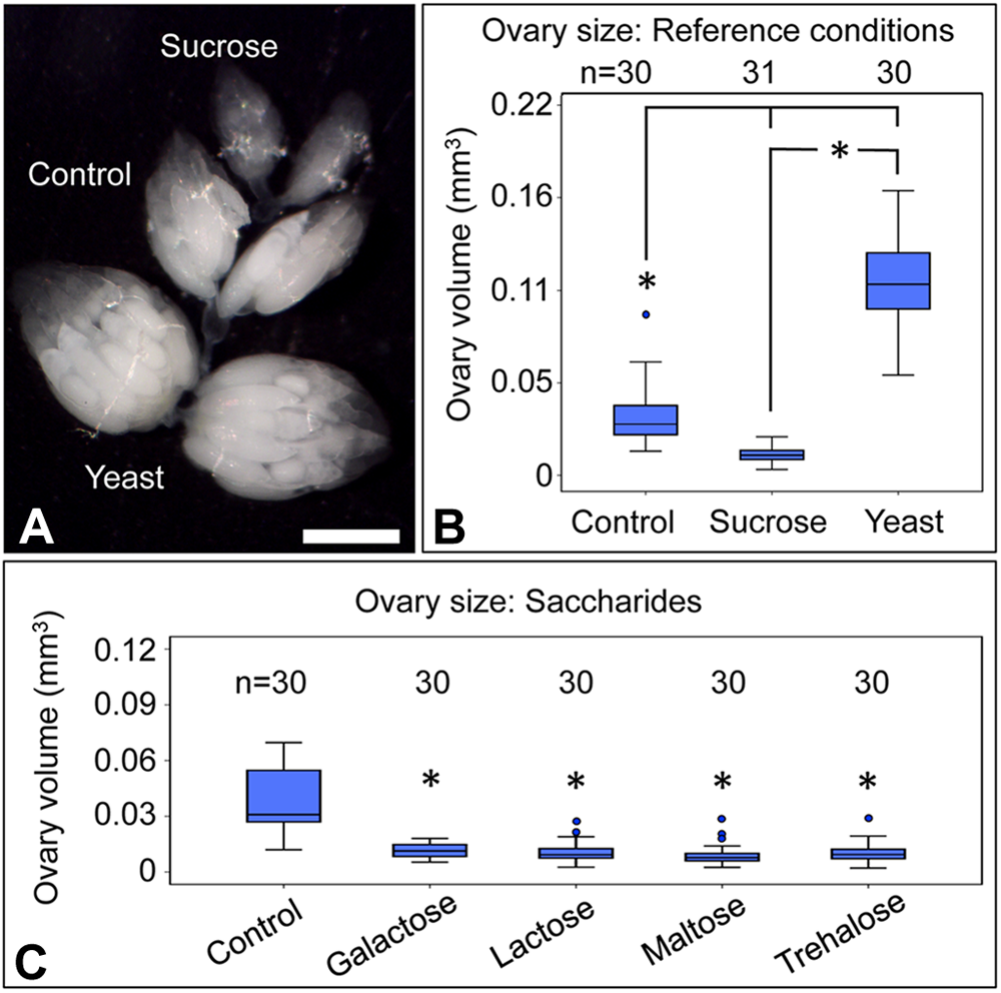
**Ovary size response to sugar-enriched diets.** (A) Image shows examples of ovaries dissected from flies exposed to control, sucrose, and yeast. Scale bar: 0.5 mm. The graphs show quantification of ovary volume after exposure to food enriched in (B) sucrose and yeast and (C) galactose, lactose, maltose, and trehalose. Ovary pairs were dissected in the context of three biological replicates, with 5 flies dissected per condition per replicate. The size of each ovary was measured independently. The sample size (*n*) for all experimental conditions is included in the figure. Median values are displayed as the middle line within each boxplot, and the boxed areas represent the interquartile range. The box whiskers indicate minimal and maximum values of the dataset, except for the outliers which are shown as solid blue circles. Significance is indicated by asterisks, as according to Kruskal–Wallis ANOVA; *P<0.001.

*D. melanogaster* ovary size is responsive to nutritional conditions. Through apparent impacts on systemic insulin signaling, sucrose-rich diets have been shown to reduce ovary size, whereas yeast-rich diets increase it (Fig. 3A) (Geminard et al., 2009; LaFever and Drummond-Barbosa, 2005; Morris et al., 2012). Direct measurement of ovary volume in response to these diets confirms that the size changes are substantial (Fig. 3B). Ovary volumes were significantly different between control and sucrose-fed oocytes [χ^2^(2)=29.1, *P*<0.001], and control and yeast-fed oocytes [χ^2^(2)=30.2, *P*<0.001] as well as sucrose- and yeast-fed oocytes [χ^2^(2)=59.3, *P*<0.001] (Fig. 3B). As dietary sucrose and yeast exert opposite impacts on ovary volume and *Wolbachia* titer, these data open the possibility that oocyte titer reflects ovary size.

To further assess the relationship between ovary size and oocyte *Wolbachia* titer, ovary volume was assessed across sugar-enriched dietary conditions. This analysis indicated consistently small ovary volumes for galactose-, lactose-, maltose- and trehalose-fed oocytes [χ^2^(4)≥57.0, *P*<0.001 for all] (Fig. 3C). As these conditions significantly elevated oocyte *Wolbachia* titers (Fig. 2E,F), this outcome is consistent with oocyte *Wolbachia* titer as an inverse correlate of ovary size. Furthermore, median ovary volumes ranged from 25% of control in the maltose condition, to 36% of the control in the galactose condition (Fig. 3C). This is analogous to size reductions seen in sucrose-fed ovaries (39% of the control) (Fig. 3B). Taken together, the data suggest that sugar-enriched diets generally lead to ovary size reduction.

### Sweet tastants affect ovary size, but not oocyte *Wolbachia* titer or oocyte size

**Fig. 4.**
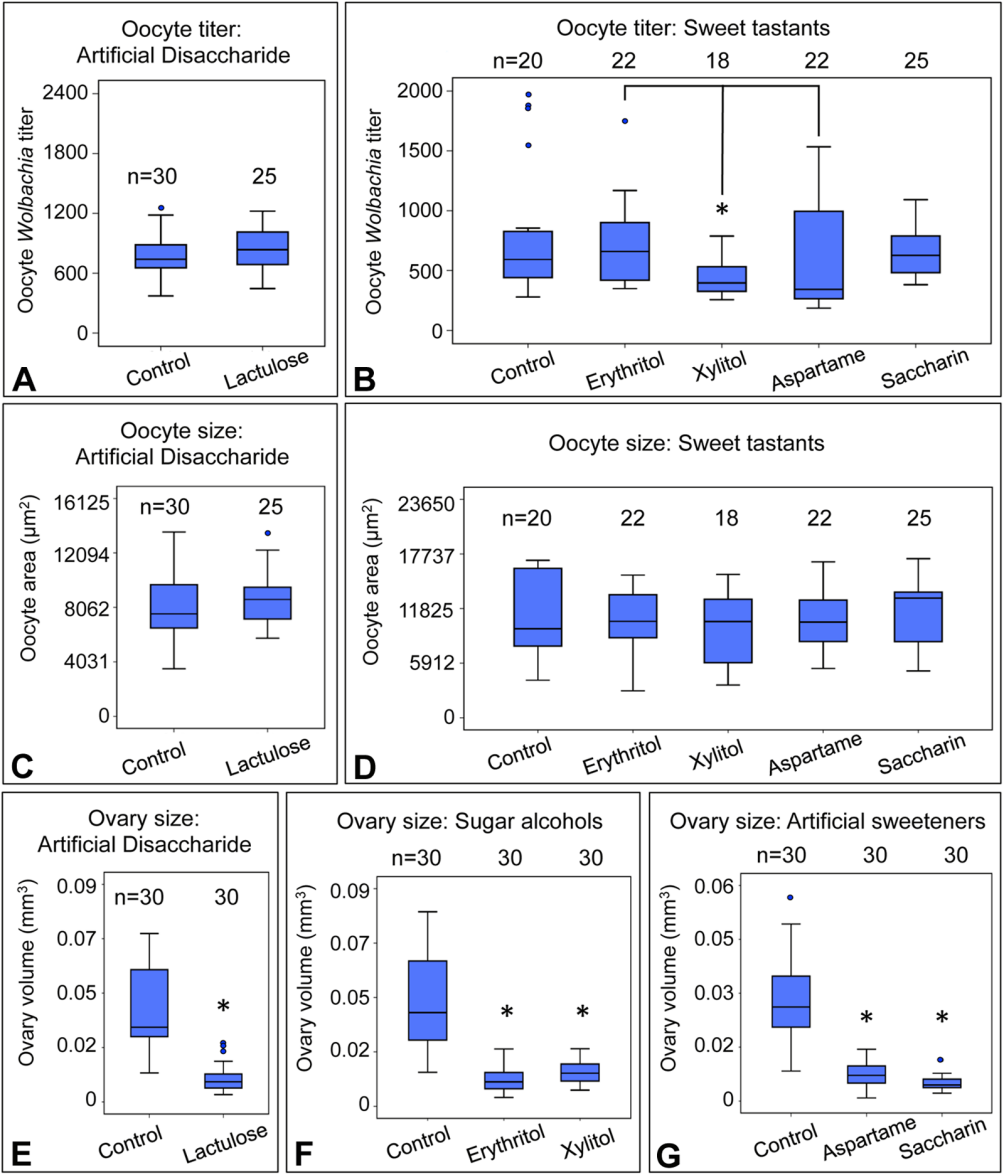
**Analysis of conditions enriched in artificial- and non-saccharide sweet tastants.** Oocyte *Wolbachia* titer is shown from flies exposed to diets enriched in (A) lactulose and (B) the sweet tastants erythritol, xylitol, aspartame and saccharin. Oocyte size is shown for dietary conditions enriched in: (C) lactulose and (D) erythritol, xylitol, aspartame and saccharin. For these experiments, three biological replicates were performed, with 20 flies dissected per condition per replicate. Stage 10 oocytes were selected at random for imaging by confocal microscopy, then analyzed to define oocyte *Wolbachia* titer and oocyte area. Ovary size was also measured in response to dietary conditions enriched in: (E) lactulose, (F) erythritol and xylitol, and (G) aspartame and saccharin. To perform this work, ovary pairs were dissected from three biological replicates, with 5 flies used per condition per replicate. Sizing of each ovary was measured independently. The sample size (*n*) for all experimental conditions is included in the figure. Median values are displayed as the middle line within each boxplot, and the boxed areas represent the interquartile range. The box whiskers indicate minimal and maximum values of the dataset, except for outliers, shown as solid blue circles. Significance is indicated by asterisks, as according to Kruskal–Wallis ANOVA. Significance values by panel are: (B) oocyte titer: xylitol vs erythritol: *P=0.034; xylitol vs saccharin: *P=0.040; (E-G) *P<0.001.

To test whether oocyte titer is selectively responsive to natural dietary sugars, an array of other sweet tastants was tested (Table 1; Table S1). Unlike natural sugars, the synthetic disaccharide lactulose (Fig. 1F) is reportedly indigestible by eukaryotes (Schmidl and Labuza, 2000). Flies exposed to lactulose-enriched diets did not exhibit any significant oocyte titer difference from the control [χ^2^(1)=1.57, *P*=0.211] (Fig. 4A). The impact of the sugar alcohols, erythritol and xylitol, as well as the artificial sweeteners aspartame and saccharin, were also tested (Fig. 1G-J). Oocyte *Wolbachia* titer was significantly different between xylitol and erythritol conditions [χ^2^(4)=28.9, *P*=0.034] as well as xylitol and saccharin conditions [χ^2^(4)=27.6, *P*=0.040] (Fig. 4B). However, no significant differences were evident when comparing the sweet tastant treatments against the control [χ^2^(4)≤26.5, *P*≥0.087 for all] (Fig. 4B). These data suggest that properties outside of taste recognition are responsible for sugar-driven increases in oocyte *Wolbachia* titer.

To consider the basis for the oocyte titers observed in sweet tastant conditions, oocyte and ovary sizing were also examined. No significant changes in oocyte area were detected in response to lactulose [χ^2^(1)=3.15, *P*=0.076], (Fig. 4C) nor artificial sweeteners and sugar alcohols [χ^2^(4)=1.75, *P*<0.782] (Fig. 4D). However, consistently small ovary size was detected in response to all sweet tastant treatments, with median ovary volumes ranging from 17% of the control for saccharin [χ^2^(2)=50.7, *P*<0.001] to 36% of the control for xylitol [χ^2^(2)=35.8, *P*<0.001] (Fig. 4E-G). Thus, ovary volume reduction associated with sweet tastants parallels that induced by sugar-enriched diets. However, as sugar-enriched diets elevate oocyte *Wolbachia* titer and sweet tastants do not, this indicates that oocyte *Wolbachia* titer is not specified exclusively by ovary size.

### Desiccation-associated host diet lowers oocyte *Wolbachia* titer

**Fig. 5.**
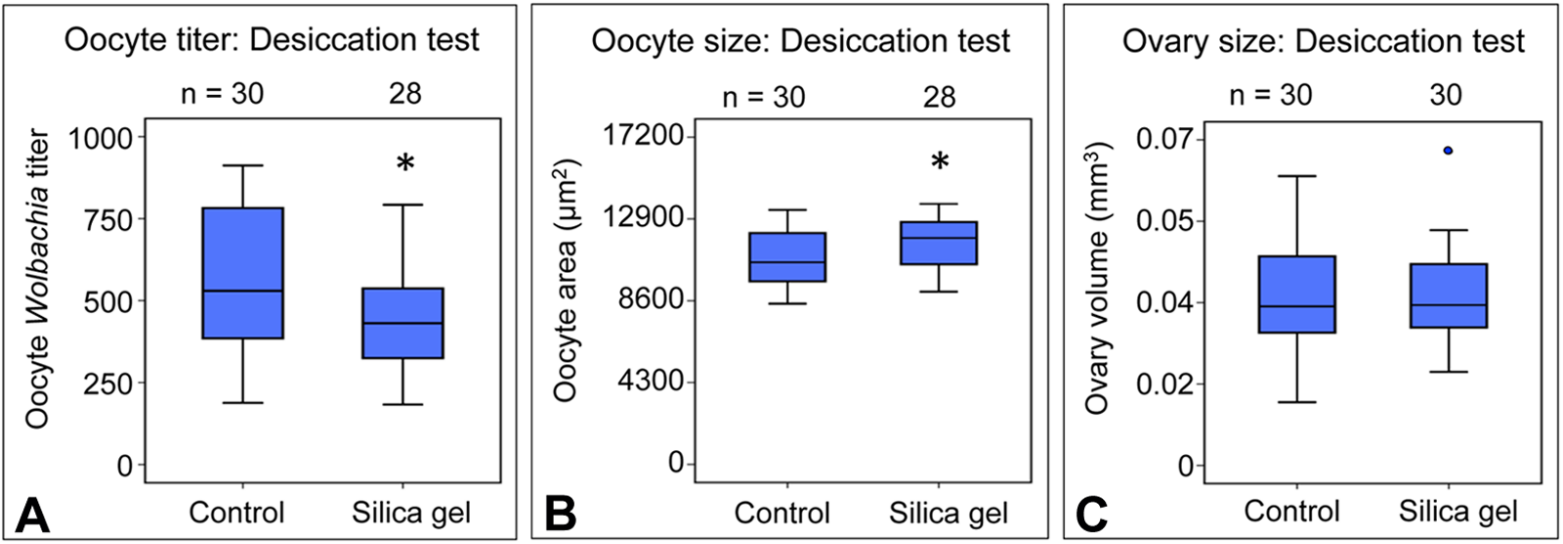
**Assessing response to desiccated food diet.** Graphical representations of (A) oocyte *Wolbachia* titer, (B) oocyte size, and (C) ovary size response to desiccated food conditions. For oocyte *Wolbachia* titer and oocyte size analysis, three biological replicates of the experiment were performed, 20 flies were dissected per condition per replicate. For ovary size analysis, ovary pairs were dissected from 15 flies total, 5 flies per replicate. Each ovary was measured independently. The sample size (*n*) for all experimental conditions is included in the figure. Median values are displayed as the middle line within each boxplot, and the boxed areas represent the interquartile range. The box whiskers indicate minimal and maximum values of the dataset, except for outliers, shown as solid blue circles. Significance is indicated by asterisks, as according to Kruskal–Wallis ANOVA. Significance values by panel are: (A) oocyte titer: control vs silica gel: *P*=0.026; (B) oocyte size: control vs silica gel: *P*=0.043.

To further investigate the basis for sugar-associated oocyte titer increases, candidate hypotheses were pursued. During food preparation, the sugar solutions were distinctively thick in consistency compared to other treatments. The apparently hygroscopic properties of the sugars opened the possibility that they may act as a desiccant after ingestion. To test the impact of desiccation on oocyte *Wolbachia* titer, flies were exposed to standard fly food containing dehydrating silica gel in a 2:1 volumetric ratio. After 3 days of exposure, samples were examined. Analysis of oocyte *Wolbachia* titer indicated that fewer *Wolbachia* were carried by the silica gel condition, with the median oocyte titer value at 81% of the control [χ^2^(1)=4.95, *P*=0.026] (Fig. 5A). Oocyte size assessment indicated that oocyte area was significantly larger in the silica gel condition, with the median area value at 112% of the control [χ^2^(1)=4.09, *P*=0.043] (Fig. 5B). No differences in ovary size were observed between control and silica gel conditions [χ^2^(1)=0.056, *P*=0.813] (Fig. 5C). These data suggest that desiccation impacts on *Wolbachia* and oogenesis are entirely distinct from that of the dietary sugars. Thus, desiccation is not responsible for the titer-increasing effects of dietary saccharides.

### Dietary sugars differentially rescue dietary yeast impact on oocyte *Wolbachia* titer

**Fig. 6.**
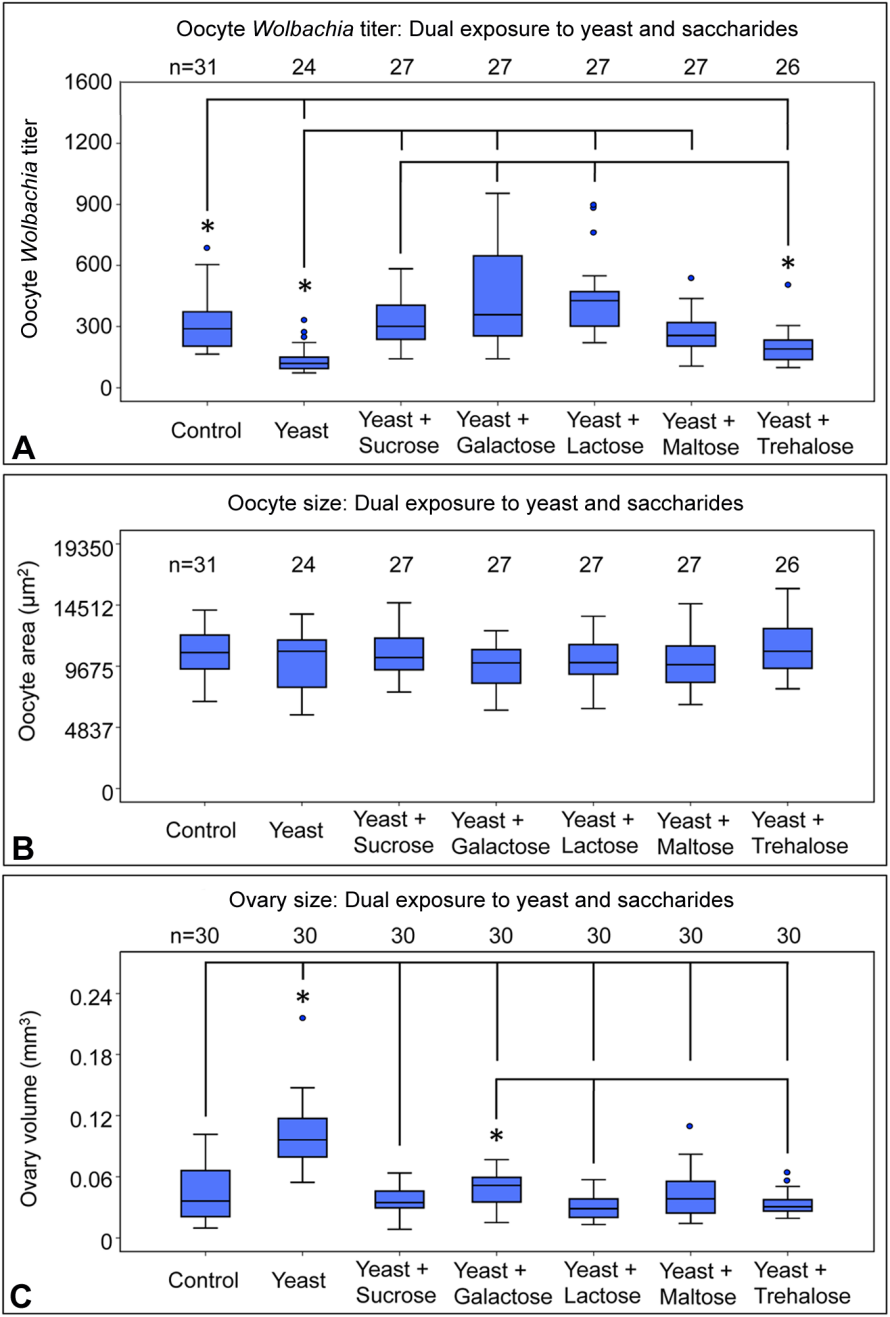
**Assessing impact of diets dually enriched in yeast and natural saccharides.** The sugars co-administered with yeast included: sucrose, galactose, lactose, maltose and trehalose. Graphs represent changes in quantification of (A) oocyte *Wolbachia* titer, (B) oocyte area, and (C) ovary volume in response to dual feeding conditions. For oocyte *Wolbachia* titer and oocyte size analyses, three biological replicates of the experiment were performed, 20 flies were dissected per condition per replicate. Stage 10 oocytes were selected randomly for confocal microscopy imaging, then followed up for quantification. For assessment of ovary size, ovary pairs were dissected from 15 flies total per condition, 5 flies per replicate. Each ovary was measured independently. The sample size (*n*) for all experimental conditions is included in the figure. Median values are displayed as the middle line within each boxplot, and the boxed areas represent the interquartile range. The box whiskers indicate minimal and maximum values of the dataset, except for outliers, shown as solid blue circles. Significance is indicated by asterisks, as according to Kruskal–Wallis ANOVA. Significance values by panel are: (A) Oocyte titer: *P<0.001 with the exception of: control vs yeast + trehalose: *P*=0.021; yeast vs yeast + maltose: *P*=0.002; and yeast + trehalose vs yeast + sucrose: *P*=0.008. (C) Ovary size: *P<0.001 with the exception of: yeast + galactose vs yeast + lactose: *P*=0.005; and yeast + galactose vs yeast + trehalose: *P*=0.035.

Another possibility is that titer-elevating sugars generally affect oocyte *Wolbachia* titer through impact on core nutritional signaling processes. It was previously demonstrated that dietary yeast and sucrose exert opposite effects on oocyte *Wolbachia* titer in an insulin-dependent manner (Serbus et al., 2015). If dietary sugars are generally antagonistic to insulin signaling, one possibility is that they will rescue the impact of dietary yeast on oocyte *Wolbachia* titer. To test this, flies were exposed to diets dually enriched in yeast and dietary saccharides. An array of responses was evident (Fig. 6A). No dual feedings of yeast and sugar elevated oocyte titer significantly above control levels. Relative to the control, oocytes exposed to diets enriched in either yeast or yeast+trehalose showed significant depletion of *Wolbachia* [χ^2^(6)=75.5, *P*<0.001 and χ^2^(6)=47.9, *P*<0.021, respectively]. Yeast-fed oocytes showed the overall lowest titer levels, differing significantly from all dual yeast-sugar feeding conditions [χ^2^(6)≥59, *P*≤0.002 for all] except yeast+trehalose [χ^2^(6)=27.5, *P*=1.000]. The yeast+trehalose condition displayed significantly lower oocyte *Wolbachia* titer as compared to dual feedings of yeast+sucrose, galactose, or lactose [χ^2^(6)≥53.6, *P*≤0.008 for all] (Fig. 6A). Thus, the yeast+trehalose oocyte titer profile paralleled many of the outcomes associated with exposure to dietary yeast alone. These data overall indicate that dietary sucrose, galactose, lactose and maltose rescue oocyte titer suppression by dietary yeast, whereas dietary trehalose does not.

### Dietary sugars consistently rescue yeast-driven ovary enlargement

To investigate the basis for differential oocyte *Wolbachia* titer responses to yeast+sugar diets, sizing controls were also performed. No significant differences in oocyte area were observed between any of the feeding conditions used [χ^2^(6)=9.03, *P*=0.172] (Fig. 6B). By contrast, ovary size was responsive to nutrient-altered diets. Ovary volumes in all yeast+sugar feeding conditions were significantly lower than in the yeast-fed condition [χ^2^(6)≥67.5, *P*<0.001 for all cases] (Fig. 6C). Furthermore, no significant ovary volume differences were observed between the control and any of the yeast+sugar dual feeding conditions [χ^2^(6)≤36.8, *P*≥0.397 for all]. This indicates that dietary sugars consistently rescued yeast-driven ovary enlargement. Some variation was detected in the extent of ovary size modification by dual yeast+sugar diets. Ovary volumes in the yeast+galactose condition were distinguished as significantly greater than the yeast+lactose condition [χ^2^(6)=57.6, *P*<0.005] and the yeast+trehalose condition [χ^2^(6)=49.3, *P*<0.035] (Fig. 6C). As these trends do not parallel oocyte titer outcomes in a consistent manner, the implication is that dietary sugars affect ovary development and *Wolbachia* colonization dynamics through mechanisms that are at least partially independent.

## DISCUSSION

The impact of diverse dietary sugars on insulin signaling has not been fully defined in *D. melanogaster*. From the perspective of *Wolbachia* endosymbiosis, this study suggests that dietary sugars induce different classes of mechanistic responses. This work showed that *D. melanogaster* diets enriched in galactose, lactose, maltose and trehalose significantly elevated oocyte *Wolbachia* titer. As oocyte size was also unaffected by most dietary sugar treatments, all observed titer increases are interpreted to represent true elevation of bacterial quantity per oocyte and not a concentration artifact of cell size. No titer-related trends were evident in terms of reported caloric value (Table S1), gustatory preferences (Stafford et al., 2012), nor the magnitude of neural responses to single tastants (Freeman et al., 2014). These outcomes are most readily reconciled with the structural content of the sugars, as maltose and trehalose are both glucose disaccharides, and lactose contains galactose as one of its major constituents. The absence of titer-elevating effects by lactulose is consistent with a possible requirement for glucose as a constituent of titer-influencing disaccharides.

Though these sugars were selected for analysis due to their structural features, *D. melanogaster* may reasonably encounter some of these sugars in nature. Maltose is reportedly the major starch breakdown product released from chloroplasts at night (Niittayla et al., 2004; Wiese et al., 2004) and is commonly associated with starchy plant products such as grains (Halford et al., 2011). Natural exposure to trehalose is less likely, as it is carried at low levels in higher plants, serving as a signaling cue (Grennan, 2007; Lunn et al., 2014). Galactose is a core component of raffinose-containing oligosaccharides that are widespread in higher plants (Sengupta et al., 2015) and carried within dozens of fruits and vegetables (Gross and Acosta, 1991). Though lactose is not expected to appear in a natural *D. melanogaster* diet, one highly speculative possibility is that lactose digestion by microbes occupying the food vial or gut microbiome releases galactose, ultimately inducing titer responses. Future analyses of sugar uptake by *D. melanogaster* are needed to further inform the relevance of ingested doses. At this time, only two (Meyer et al., 2011; Wang and Wang, 1993) of the 26 predicted sugar transporters in *D. melanogaster* (Adams et al., 2000; Marygold et al., 2016) have been characterized. However, 17 of the predicted transporter proteins are expressed by females and detected in digestive tissues, and are thus potentially relevant for consideration (Table S2).

Another finding of this study was that ovary size responses to dietary cues did not consistently correspond to oocyte titer changes. As ovary size and oocyte *Wolbachia* titer are oppositely affected by sugar- and yeast-enriched diets, the simplest interpretation is that spatial re-allocation of *Wolbachia* within the ovary is responsible for increased oocyte titers. This hypothesis invokes horizontal *Wolbachia* invasion between cells of the ovary as influential in oocyte colonization by *Wolbachia*. Invasion has been reported to contribute to *Wolbachia* colonization of the distal tip of the *Drosophila* ovary (Toomey et al., 2013), early stages of *Drosophila* oogenesis (Casper-Lindley et al., 2011) and ovarian cells of mosquitoes and nematodes (Hughes et al., 2012; Landmann et al., 2012). Sugar alcohol and artificial sweetener treatments deviated from this invasion paradigm, as their reductions of ovary size were not paralleled by oocyte titer increases. Thus, ovary size may modulate oocyte colonization by *Wolbachia* titer in some cases, but the data argue against ovary size as a binary predictor of oocyte titer. One possibility is that changes in bacterial loading and replication in the germline offset changes in the extent of horizontal invasion. Reduced oocyte titers seen in xylitol-fed oocytes suggest that antibiotic properties of this sugar alcohol (Katsuyama et al., 2005; Renko et al., 2008) may exaggerate such tendencies. Another possibility is that dietary saccharides and sweet tastants each alter ovary size in a different manner. Ovary size reflects the number of productive ovarioles as well as the rates of egg chamber production, egg development and egg laying by each female (King, 1970). At this time, it cannot be ruled out that the physical basis for small ovary size may differ between saccharide and sweet tastant conditions.

This study further sought to address the mechanistic basis for saccharide impacts on oocyte *Wolbachia* titer. One possibility was that the concentrated sugar additives may increase oocyte *Wolbachia* titer as an indirect consequence of ovarian responses to desiccation. However, dietary desiccation tests showed reduction of oocyte *Wolbachia* titer rather than an increase, suggesting that sugar-based titer responses are unrelated to hydration. Another formal possibility is that *Wolbachia* responsiveness to dietary sugars is due to uptake of these sugars and/or their derivatives after ingestion. Though *Wolbachia* are predicted to encode a single hexose phosphate transporter, WD_0619, homologous to GlpT/PgpT/UhpT of *Escherichia coli* (Fann and Maloney, 1998; Kadner et al., 1992), there is no information to suggest the *Wolbachia* homolog of this transporter is sufficiently permissive to take up the diverse dietary sugars analyzed in this study.

The impact of dietary sugars on oocyte *Wolbachia* titer is currently best explained through nutritional impacts on the host. Dietary yeast is expected to activate multiple nutritional signaling branches, including insulin signaling, that converge upon activation of the mTORC1 kinase complex (Geminard et al., 2009; Teleman, 2010). Prior work showed that chemical inhibition of mTORC1 increased oocyte *Wolbachia* titer analogous to dietary sucrose, while loss of mTORC1 suppression lowered oocyte titers (Serbus et al., 2015). These findings and others implicated insulin as a suppressor of oocyte *Wolbachia* titer and inherently suggested that yeast-associated phenotypes should be ameliorated by dietary sugars. This study showed that dietary sugars did suppress yeast-associated ovary enlargement across the board, consistent with such a prediction. However, oocyte *Wolbachia* titers showed a range of responses, with trehalose exerting no impact on yeast-driven titer depletion, whereas sucrose, galactose, lactose and maltose restored oocyte titer to control levels. The disparity is surprising, as trehalose-enriched diets elicited the largest recorded oocyte titer increase to date. Examination of ovary size further indicated that galactose rescue of yeast-driven ovary enlargement was significantly less effective than lactose and trehalose. These findings suggest that saccharide treatments, all singly capable of elevating oocyte *Wolbachia* titer, may exert distinct functional impacts on oocyte *Wolbachia* titer. Integrated quantitative analyses will play an important role going forward in elucidating the mechanisms of oocyte colonization by *Wolbachia.*

## MATERIALS AND METHODS

### Fly food preparation

The standard food used in this study is based upon a recipe by the Bloomington Drosophila Stock Center (http://flystocks.bio.indiana.edu/Fly_Work/media-recipes/bloomfood.htm). Our fly food was prepared in large batches that consisted of 20 liters water, 337 g yeast, 190 g soy flour, 1325 g yellow corn meal, 96 g agar, 1.5 liters Karo light corn syrup and 94 ml propionic acid. This standard food was used as a base for all nutrient-altered foods that were prepared in this study (Table S3). The sugar-enriched foods were prepared by first making a stock sugar solution of 20 g sugar in 10 ml ddH_2_O, solubilized with rounds of 15 s in the microwave and then stirring, repeated until the sugar dissolved. 1.5 ml amounts of these sugar solutions were immediately mixed with 3.5 ml of melted standard food. As aspartame, erythritol, saccharin, and xylitol were not uniformly soluble, the sweetener-enriched foods were generated through direct addition of powder equivalents directly into 5 ml of melted standard food to a final concentration of 1 M (Table S3). Yeast-enriched food was prepared by mixing 1.5 ml of heat-killed yeast paste into 3.5 ml melted standard food. Dually enriched food was prepared through addition of 1.5 ml sugar solution and 1.5 ml heat-killed yeast to 2 ml standard food. Desiccated food was prepared by addition of 2.5 g silica gel (roughly 2.5 ml volume) to vials containing 5 ml standard food (Table S3). To ensure homogeneous suspensions of nutrient-altered diet preparations, all food vials were immediately transferred to an ice bucket to be cooled with additional stirring every 10 min until the food completely solidified. Kimwipe strips were inserted into the food to wick away excess moisture.

All feeding experiments were done using flies of the genotype *w; Sp/Cyo; Sb/Tm6B*, reared on standard food and in a controlled, 25°C environment. This stock carries the *w*Mel *Wolbachia* strain as confirmed previously (Christensen et al., 2016). 0−24-hour-old adult flies were selected at random and transferred into new bottles of standard food and aged for 2 days. Then flies were transferred to vials of nutrient-altered food and incubated for 3 days. Controls were run in parallel with all treatment conditions in all experiments.

### Tissue staining, imaging, and analysis

Ovarian tissues were dissected in PBS and fixed in 2% formaldehyde for 20 min as previously described (Serbus et al., 2015). The ovaries were rinsed with PBS-Triton 0.1% (PBS-T), incubated overnight in 10 mg ml^−1^ RNAse, and rinsed extensively with PBS-T the next day. Then tissues were infused with 70% glycerol that contained 0.015 mg ml^−1^ propidium iodide, and mounted on a slide. All replicates were imaged by laser scanning confocal microscopy on either Leica SP2 or an Olympus FV1200 confocal microscope at 63× magnification with 1.5× zoom. The Z-height of oocytes on each slide was standardized against the control slide for each replicate. Z-series images were acquired from randomly selected stage 10 egg chambers at 1.5 μm intervals. Uniform intensity settings were applied to all egg chambers imaged in each replicate.

To quantify oocyte *Wolbachia* titer, stacks of confocal images were examined to identify the deepest possible focal plane where *Wolbachia* are clearly visible across all samples of the replicate (Serbus et al., 2015). Images were manually processed in Photoshop to remove extraneous signal outside the oocyte, and remaining oocyte puncta were quantified using the Analyze Particles feature in Image J version 2.0.0-rc-43/1.51d (NIH). Thus, these data quantify the *Wolbachia* titer carried within one focal plane of each oocyte. This has been verified as a representative measure for comparing oocyte *Wolbachia* titer between different conditions (Serbus et al., 2011). Three or more experimental replicates were performed for all treatment conditions examined. Significance of differences between conditions was determined by ANOVA analysis of the raw data.

For measurement of oocyte area, the same representative oocyte focal planes used for *Wolbachia* titer assessment were re-analyzed. Oocytes were manually outlined in Microsoft PowerPoint, and the resulting two-dimensional shapes flood-filled in with color. Screen shots of these ovary fill diagrams were then imported into Fiji (Image J version 2.0.0-rc-43/1.51d, NIH) for conversion into 8-bit, thresholded black and white images. The area of the ovary fill diagrams was determined in terms of pixels^2^ by the Analyze Particles function in Fiji. A scale bar was also used to calculate a pixel^2^ to micron^2^ ratio (9.3025:1) that was applied to all oocyte area data, for presentation and discussion purposes only. Statistical differences were determined through analysis of the primary data in terms of pixel^2^ units.

For measurement of ovary volume, tissues were dissected from adult flies and imaged using an AmScope MD500 5.0 megapixel digital Camera mounted upon a Jenco ST-F803 dissection microscope set at 1× magnification. The pixel length and width of each ovary was assessed with the ‘Measure’ tool in Fiji. These values used to approximate ovary volume using the standard ellipsoid formula for volume; *V*=4 3*πabc*^−1^, where *a*=½ the length and *b* and *c*=½ the width. Three biological replicates were assessed for all treatment conditions. The area of a reference object was measured to determine the pixel to mm ratio (148.62:1) appropriate for describing the volumetric data. This conversion was applied in the context of presentation and discussion purposes only. All statistical analyses of ovary volumes were based upon primary data in terms of the pixel^3^ units.

### Statistical analysis

All data in this study were analyzed with the IBM SPSS statistics program, v.23. The descriptive statistics function was used to analyze the distribution of the data. According to the residuals as well as metrics such as skewedness and kurtosis, the current data did not fit a normal distribution. However, the data met the assumptions of the two-tailed Kruskal–Wallis ANOVA, and thus this test was systematically applied for data analysis. Variation within each experimental condition is indicated by boxplot format used to graphically display the data. Post hoc data presented here were generated by SPSS as standard outputs of the analysis, including the adjusted *P*-values reported throughout the manuscript. Though subtle differences in oocyte titer, oocyte area and ovary volume may not be detected by this analysis framework, the data empirically demonstrated that the sample ‘*n*’ for these experiments was sufficient to identify clear cases of significance.
